# Piperonylic acid stimulates keratinocyte growth and survival by activating epidermal growth factor receptor (EGFR)

**DOI:** 10.1038/s41598-017-18361-3

**Published:** 2018-01-09

**Authors:** Dohyun Lee, Jinsun Lim, Kyung-Chul Woo, Kyong-Tai Kim

**Affiliations:** 1 Department of Life Sciences, Pohang University of Science and Technology, Pohang, 37673 Republic of Korea; 2 Division of Integrative Biosciences and Biotechnology, Pohang University of Science and Technology, Pohang, 37673 Republic of Korea; 3 Newlife Cosmetics R&D Center for Skin Science, Gyeongsan, Gyeongbuk Republic of Korea

## Abstract

Epidermal growth factor (EGF) stimulates cell growth, proliferation, and survival. The biological benefits of EGF have been utilized in medical uses for improving wound healing as well as in today’s skin cosmetics. EGF has been found in urine, saliva, milk, and plasma, but its efficient isolation remains a difficult task. With technical advances, recombinant protein purification technique has been used for EGF production. However, the recombinant EGF is still expensive and keeping it with stable activity is difficult to be used widely. Thus, a molecule that can mimic the EGF activity would be a useful alternative of EGF. Herein, we have discovered that a natural small molecule piperonylic acid shows EGF-like activity in HaCaT keratinocytes. Piperonylic acid induced EGF receptor (EGFR) activation and resulted in serial activation of the downstream modulators. The activated signaling pathway eventually up-regulated gene expression of *egr-1*, *c-fos*, *c-jun*, and *c-myc*, which are involved in cell growth and survival. Moreover, piperonylic acid showed promoting role in keratinocyte growth and survival from UVB-induced cellular damages. This study has revealed the EGF-like activity of piperonylic acid and proposed that the piperonylic acid could be a promising component for skin wound healing agents or cosmetic ingredient.

## Introduction

The epidermal growth factor receptor (EGFR) signaling pathway is one of the most important pathways that stimulate epidermal cell growth, proliferation and survival^1^. The molecular feature of the EGFR signaling pathway is the activation of MAPK/ERK, PI3K/AKT, and JAK/STAT pathways^2,3^. Their activation eventually promotes gene expression involved in cell growth and survival^2,3^. Because this signal transduction is mitogenic and pro-survival, epidermal growth factor (EGF), the ligand of EGFR, has been used as a cutaneous wound healing agent for burns and severe ulcers^4–6^. Furthermore, these days, EGF has emerged as an effective skin cosmetic ingredient as its biological activity and the dermal aging mechanism have been unveiled^7–10^.

The 53-amino acid-long human EGF has been isolated from human urine, saliva, milk, and plasma, but the yield was too low^11^. To solve the low yield problem, chemical synthesis or recombinant protein purification techniques have been developed and applied to obtain higher yield of EGF^12,13^. However, the cost for synthesis or purification is still expensive and the quality control in active state made it rather difficult to be used widely. Therefore, a stable and safe molecule that can mimic the action of EGF would be useful as an EGF alternative. In the present study, we have made efforts to discover active natural small compounds for the aforementioned purpose and have found a pepper derived EGF-like small molecule that can activate EGFR and its downstream signaling.

Pepper is one of the widely used spices in the world. It has also long been applied to medicinal purposes since ancient times and was reported to show multiple biological activities such as anti-cancer, anti-inflammatory, anti-bacterial, and anti-oxidant activities^14–17^. Piperonylic acid is a natural small compound isolated from black (Piper nigrum L.) and long (Piper longum L.) peppers^18^. Two recent studies reported that piperonylic acid showed lipoxygenase and intestinal α-glucosidase inhibitory activities as well as anti-oxidant activity^19,20^. However, its additional biological activity is still poorly understood. Herein, we report a novel biological property of piperonylic acid, which is useful for the stimulation of keratinocyte growth and survival signaling pathways via EGFR activation.

## Results

### EGFR was activated by piperonylic acid

**Figure 1:**
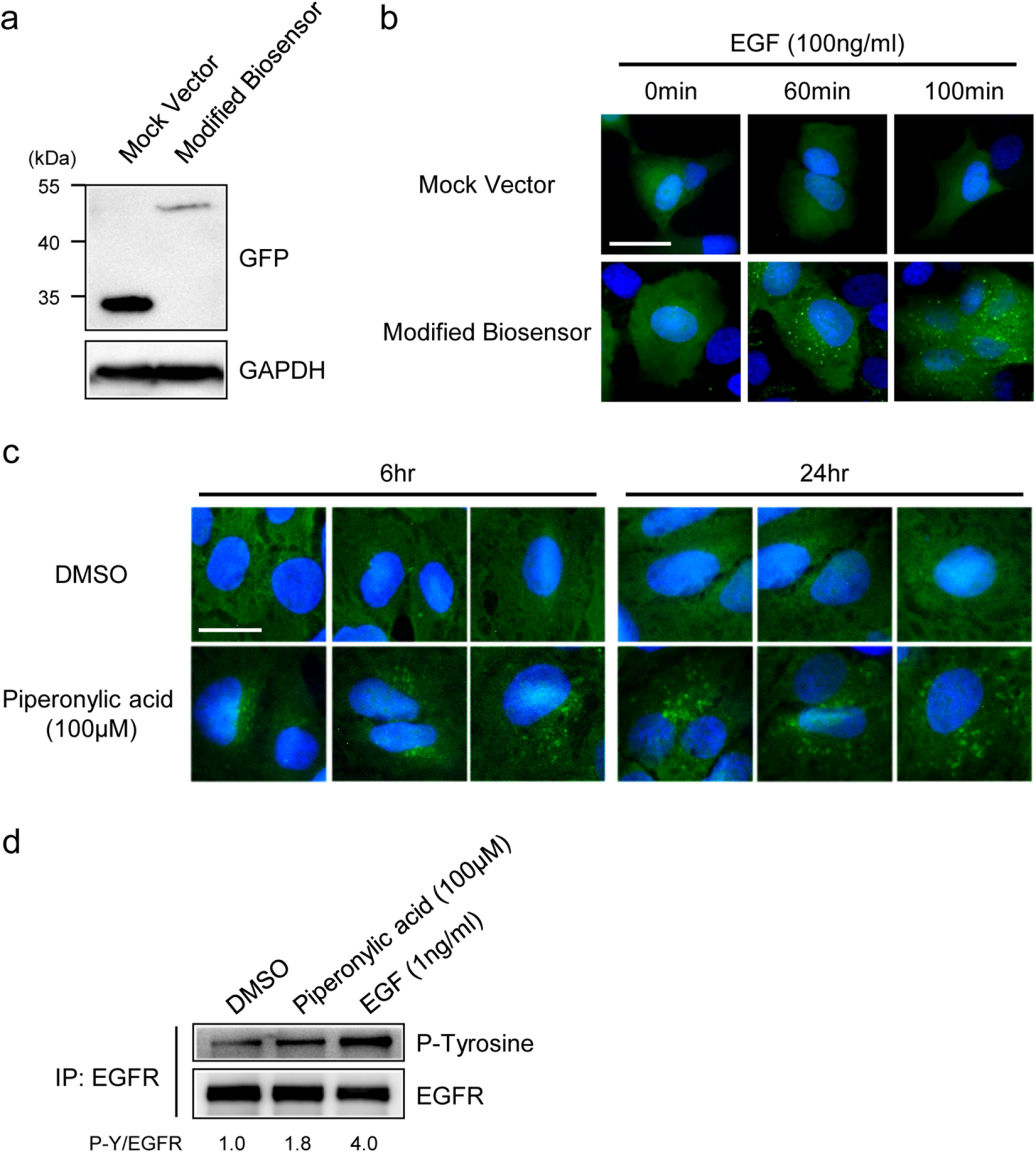
EGFR was activated by piperonylic acid. (**a**) The modified EGFP-SH2 biosensor protein was well expressed with the expected size in transfected A549 cells (expected molecular weight of a duplicate of SH2 domains is 20 kDa). (**b**) The modified biosensor system detected EGFR activation shown in bright green fluorescent spots. (**c**) The modified biosensor system recognized EGFR activation upon piperonylic acid treatment. (**d**) Tyrosine phosphorylation of EGFR was increased by piperonylic acid treatment (10 minutes). Scale bar, 50 μm. Blue fluorescence represents nucleus.

To discover small compounds that were active on EGFR, we modified and utilized a biosensor system that was developed as previously reported^21–23^. The modified biosensor system has two EGFP fused SH2 domains that is under the control of *EF1α* promoter (Supplementary Fig. S1a). The modified biosensor protein was well expressed with the expected size in transfected cells and its fluorescence was well distributed in the cytosol before EGF stimulation (Fig. 1a and b). We, then, treated 100ng/ml of EGF to the cells transfected by the modified biosensor vector or mock vector to validate the modified biosensor system. The bright green fluorescent spots were detected well within an hour and accumulated as time passed (Fig. 1b, Supplementary Fig. S1b). Moreover, just like the original biosensor system, the modified biosensor was also quite sensitive to EGF compared to other Grb2 mediating growth factors^21^ (Supplementary Fig. S1c). Based on these results, we demonstrated that the modified biosensor system was made well to recognize EGF or EGF-like acting molecules.

Next, we performed pilot screening assays using the modified biosensor system and found that piperonylic acid was appeared to be positive. When 100 μM of piperonylic acid was treated to biosensor transfected cells, the EGFR activation was detected 6 hours post treatment and was accumulated at 24 hours after treatment (Fig. 1c). To validate the activation of EGFR, we immunoprecipitated cell lysate using anti-EGFR antibody and analyzed the levels of the tyrosine phosphorylation on EGFR in HaCaT cells. HaCaT cells are keratinocytes that express robust EGFR (Supplementary Fig. S2a). When 100 μM of piperonylic acid was treated for 10 minutes, the tyrosine phosphorylation of EGFR was increased compared to DMSO control but not as much as when compared with 1ng/ml of EGF treatment (Fig. 1d). This suggests that piperonylic acid resulted in tyrosine phosphorylation of EGFR but the activity was lower than EGF. Another type of growth factor receptor, fibroblast growth factor receptor 2 (FGFR2), was not activated by piperonylic acid treatment (Supplementary Fig. S2b), suggesting that piperonylic acid was relatively selective to EGFR.

We additionally examined whether or not the piperonylic acid can interact with EGFR. We performed a pull-down assay using HaCaT cell lysate with piperonylic acid-coupled Sepharose-4B (Piperonylic acid-4B) or control-4B beads. It was found that EGFR was bound to piperonylic acid-4B but the binding was weakened when free piperonylic acid was added for competition (Supplementary Fig. S3a). These data suggest that the interaction of piperonylic acid with EGFR might participate in facilitating EGFR activation.

### Piperonylic acid-induced EGFR activation resulted in activation of ERK and AKT

**Figure 2:**
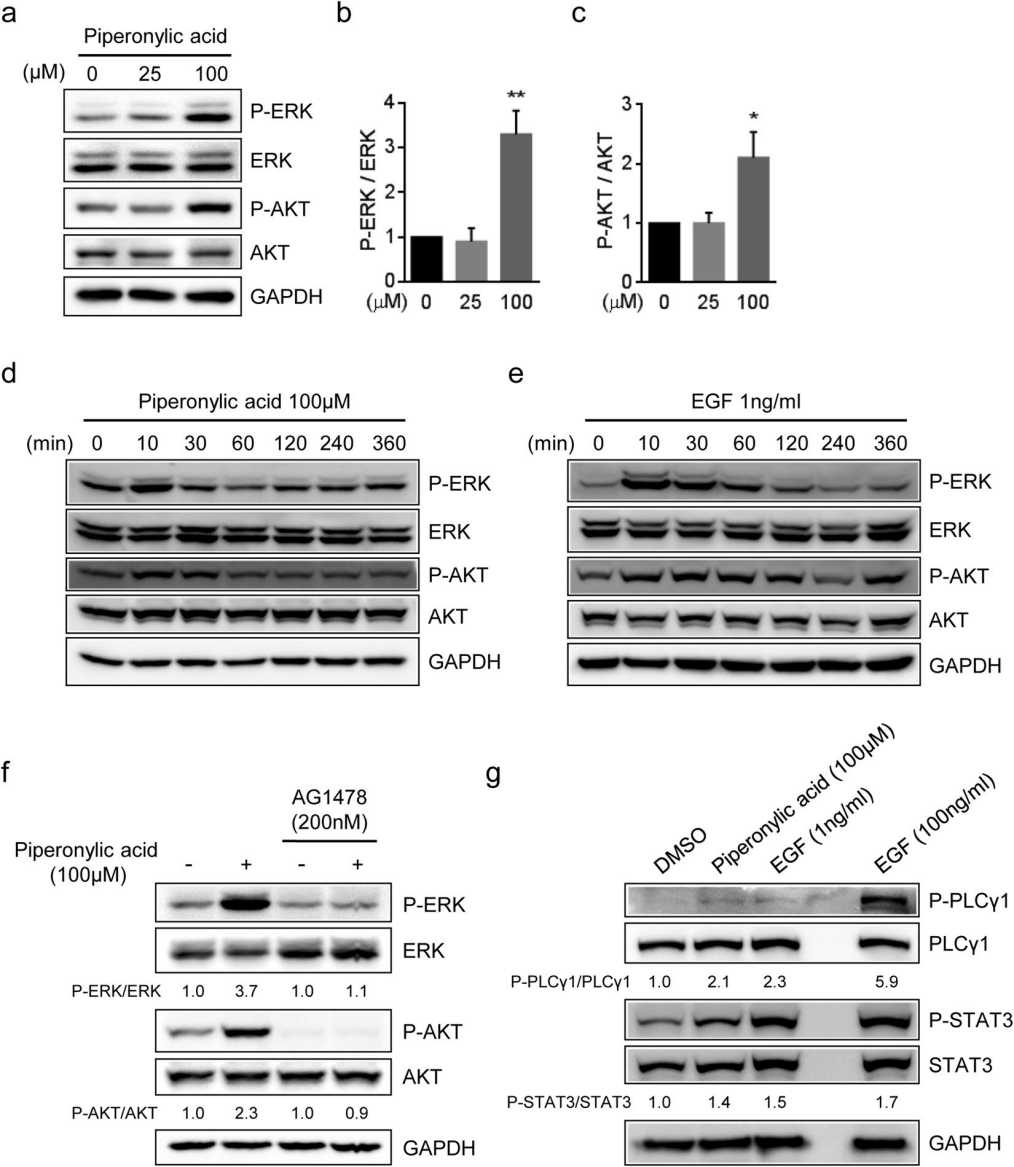
Piperonylic acid-induced EGFR activation resulted in activation of the downstream modulators of EGFR. (**a**) Both ERK and AKT were activated by 10 minutes treatment of indicated amounts of piperonylic acid in HaCaT cells. (**b**,**c**) Quantification of bands densities of P-ERK and P-AKT, respectively (n = 3). All data are presented as mean ± SD. Unpaired t-test. *p < 0.05, **p < 0.01. (**d**,**e**) Time dependent modulation of ERK and AKT phosphorylation by 100 μM of piperonylic acid or 1ng/ml of EGF, respectively, in HaCaT cells. (**f**) Piperonylic acid-induced ERK and AKT activation was blocked by EGFR antagonist AG1478. (**g**) PLCγ1 and STAT3 were activated by treatment of HaCaT cells with 100 μM of piperonylic acid for 10 minutes.

ERK and AKT are well-known downstream modulators of EGFR signaling pathway^24^. To examine the effect of piperonylic acid on the activation of ERK and AKT, we treated 25 μM and 100 μM of piperonylic acid to HaCaT cells for 10 minutes and examined the phosphorylation of ERK and AKT. As a result, when compared with DMSO control, the phosphorylation of ERK and AKT by 100 μM of piperonylic acid was increased by 3.3 and 2.1 fold, respectively (Fig. 2a–c). We then investigated the ERK and AKT activation by piperonylic acid upon treatment time. Both ERK and AKT phosphorylation was increased immediately after 100 μM of piperonylic acid treatment but down-regulated as time passed, which was similar to the activation pattern induced by 1ng/ml of EGF (Fig. 2d and e).

Next, to verify that the piperonylic acid-induced ERK and AKT activation was dependent on EGFR activation, we pre-incubated cells with an EGFR antagonist AG1478 for 10 minutes and then treated with 100 μM of piperonylic acid for 10 minutes. As a result, the ERK and AKT activation was completely blocked in spite of the treatment of piperonylic acid (Fig. 2f). These data demonstrate that the piperonylic acid-induced ERK and AKT activation was due to EGFR activation.

### Piperonylic acid increased gene expression involved in cell growth and survival

**Figure 3:**
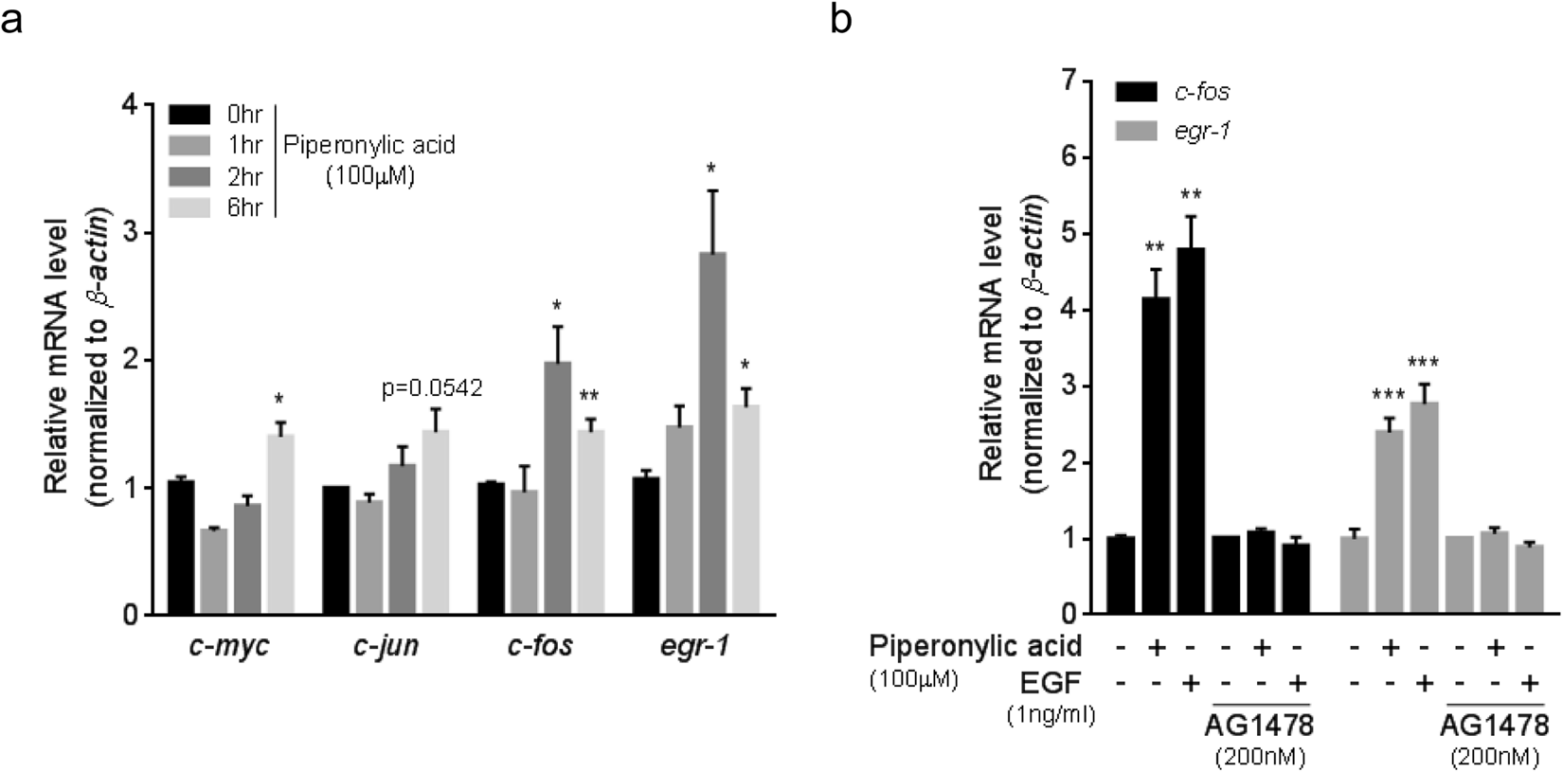
Piperonylic acid increased gene expression involved in cell growth and survival. (**a**) Time dependent gene expression changes by piperonylic acid (100 μM) treatment (n = 4). Data are presented as mean ± SEM. Unpaired t-test. *p < 0.05, **p < 0.01. (**b**) *c-fos* and *egr-1* mRNA expression by piperonylic acid or EGF (at 2 hours post treatment) in the presence or absence of AG1478 (n = 4). Data are presented as mean ± SEM. Unpaired t-test. **p < 0.01, ***p < 0.001.

The simultaneous ERK and AKT activation promotes gene expression involved in cell growth and survival^2,3,24^. *c-myc*, *c-jun*, *c-fos*, and *egr-1* are their well-known target genes regulating cell growth and survival^25–27^. We examined the expression changes of these genes upon piperonylic acid treatment. When 100 μM of piperonylic acid was treated to HaCaT cells, *c-myc*, *c-fos*, and *egr-1* mRNAs were significantly increased and *c-jun* mRNA showed tendency to increase (Fig. 3a). Consistent with the mRNA expression patterns, the protein levels of c-Fos and Egr-1 were also increased (Supplementary Fig. S4a). We, then, examined whether or not the increased *c-fos* and *egr-1* expression by piperonylic acid can be blocked by antagonizing EGFR activation. We treated 100 μM of piperonylic acid or 1ng/ml of EGF to cells for 2 hours in the presence or absence of AG1478. The expression of *c-fos* and *egr-1* mRNAs were significantly increased by both piperonylc acid and EGF, but these increments were completely diminished by AG1478 treatment (Fig. 3b). The protein levels were also shown to be consistent with the mRNA levels (Supplementary Fig. S4b). These data suggest that piperonylic acid-induced EGFR activation and its downstream signaling pathway resulted in the regulation of the gene expression involved in cell growth and survival.

### Piperonylic acid promoted keratinocyte growth and survival

**Figure 4:**
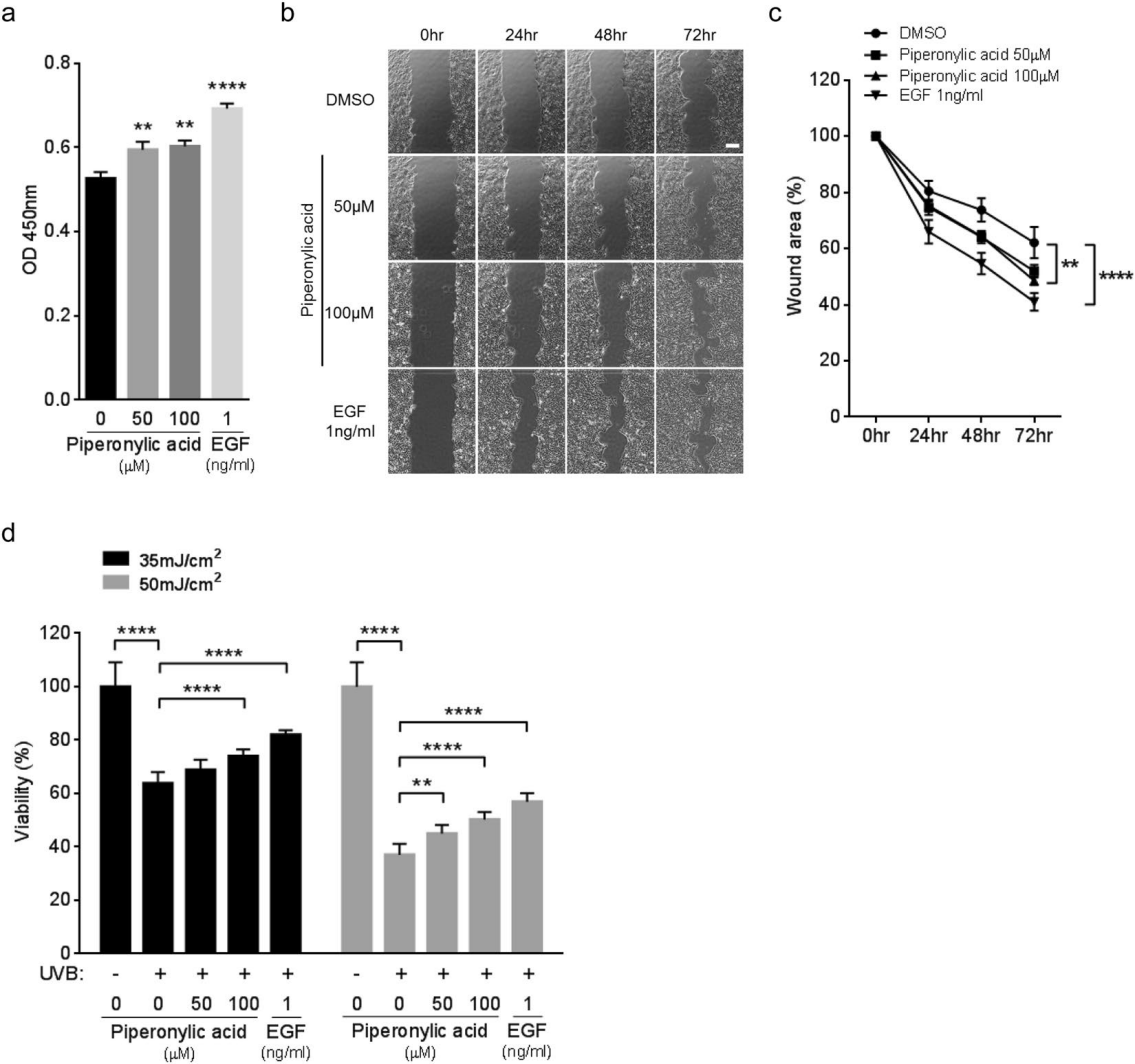
Piperonylic acid promoted keratinocyte growth and survival. (**a**) HaCaT cell growth was promoted by piperonylic acid treatment measured by 24 hour CCK-8 cell growth assays (n = 12). Data are presented as mean ± SEM. Unpaired t-test. **p < 0.01, ****p < 0.0001. (**b**) Piperonylic acid increased healing process after the scratching of the confluent HaCaT cells. Scale bar, 250 μm. (**c**) Summary of measures of the wound healing assays (n = 6). Data are presented as mean ± SEM. Two-way ANOVA followed by Tukey’s multiple comparisons test. **p < 0.01, ****p < 0.0001. (**d**) Recovery of cell viability from UVB irradiation damage (n = 12). Data are presented as mean ± SEM. One-way ANOVA followed by Tukey’s multiple comparisons test. **p < 0.01, ****p < 0.0001.

How did the changes of gene expression by piperonylic acid affect cells physiologically? To answer this question, we investigated the effects of piperonylic acid on cell growth and survival. Firstly, we performed cell growth assay with piperonylic acid. Compared to the DMSO (vehicle) treatment, 50 μM and 100 μM of piperonylic acid treatment significantly increased the growth of HaCaT cells (Fig. 4a). The 1 ng/ml of EGF treatment, which acts as a positive control, also promoted cell growth as well (Fig. 4a). We next performed wound healing assay, which is another widely adapted method for measuring cell growth and survival^28^. The wound area closure was significantly facilitated by piperonylic acid or EGF during 3 days incubation when compared with the DMSO control (Fig. 4b and c). Moreover, because HaCaT cells were skin keratinocytes, we additionally examined whether or not piperonylic acid can recover cell viability from UVB-induced damage. The UVB (280–315 nm) photon penetrates epidermal layers, where keratinocytes are the most abundant, and causes cellular damages including oxidative stress, inflammation, and DNA damages^29^. The 35 and 50 mJ/cm^2^ of UVB irradiation decreased HaCaT cell viability compared to non-irradiated cells, but the incubation with piperonylic acid after UVB irradiation significantly rescued the viability (Fig. 4d). These data suggest that the activation of EGFR signaling pathway by piperonylic acid eventually promoted HaCaT cell growth and survival and recovered cell viability from UVB-induced cellular damages.

## Discussion

**Figure 5:**
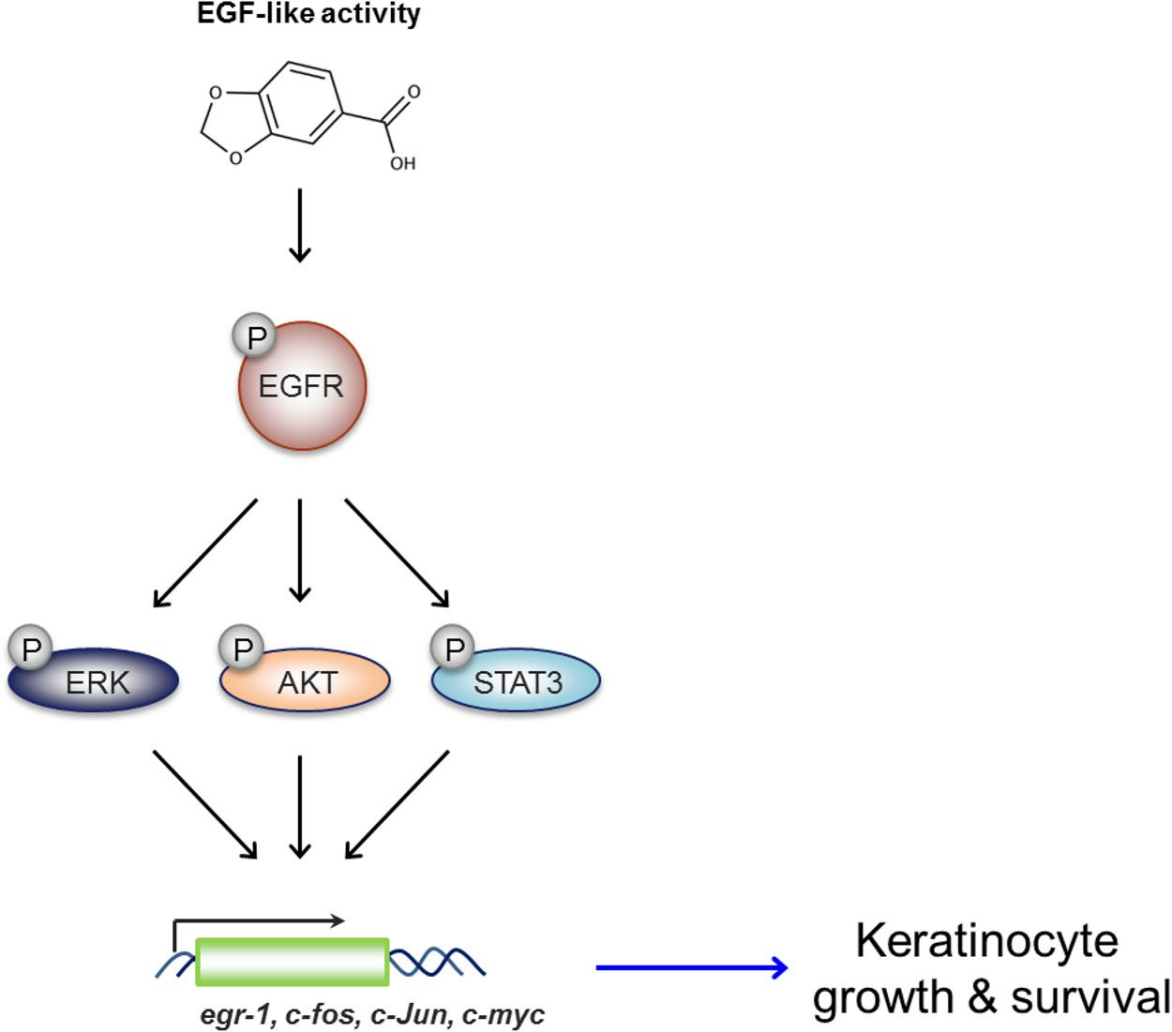
Diagram of the signaling pathway activated by piperonylic acid. Piperonylic acid induced activation of EGFR and its downstream modulators. The activated signaling pathway modulated gene expression and promoted cell growth and survival in HaCaT keratinocytes.

This study revealed the EGF-like activity of piperonylic acid. It activated ERK and AKT phosphorylation and their downstream gene expression via EGFR activation. Moreover, PLCγ1 and STAT3 activation was also accompanied with the EGF-like acitivity of piperonylic acid (Fig. 2g). The activated signaling pathway consequently resulted in the stimulation of keratinocyte growth and survival (Fig. 5).

EGF at nanomolar or subnanomolar concentration induces EGFR activation and accurately regulates signal transduction and gene expression in cells^30^. The treated concentration of EGF in this study (1 ng/ml) is equal to 156.3pM based on its molecular weight. Although the 100 μM of piperonylic acid showed significant growth and survival promotion in HaCaT cells, the effects were less than the effect by smaller molar amount of EGF (Fig. 4). Moreover, the increase of EGFR activation by 100 μM of piperonylic acid was lower than 1ng/ml of EGF (Fig. 1d). Due to the ability of piperonylic acid for activating EGFR was lower than the authentic ligand EGF, higher concentration of piperonylic acid might be inevitable to induce EGFR activation. Therefore, we assume that strong EGFR activation is considered to be beyond the capability of piperonylic acid.

One possible mechanism for EGFR activation by piperonylic acid is through generation of hydrogen peroxide. Recent studies reported that peroxide induces sulfenylation on the catalytic site of EGFR and facilitates its activation, and that some small compounds can activate EGFR through generating hydrogen peroxide^31,32^. However, 100 μM of piperonylic acid did not generate any reactive oxygen species (ROS) (Supplementary Fig. S5). Another possible mechanism is through interaction with EGFR. As EGFR interacted with piperonylic acid-coupled Sepharose-4B, the direct or indirect binding can promote EGFR activation (Supplementary Fig. S3a). In addition to the result of the pull-down assay, we found several putative binding pockets for piperonylic acid at the extracellular region of EGFR^33,34^ (Supplementary Fig. S3b). Although the predicted binding pockets for piperonylic acid were different from the EGF binding pocket^35^ (Supplementary Fig. S3c), we guess that the interaction may participate in EGFR activation somehow.

EGFR signaling pathway is important to epidermal cell growth and survival^1^, but it should be regulated properly because its excessive activation is correlated with cancer^36^. To cope with aberrant EGFR activation, cells intrinsically have down-regulation mechanisms^37–40^. In the case of the action of piperonylic acid on normal keratinocytes, the intrinsic down-regulation mechanisms seemed to function well even though its activity was quite low compared to EGF (Figs 1c and 2d). Therefore, we suppose that piperonylic acid may not alter the intrinsic down-regulation mechanisms of EGFR activation.

Although abnormal EGFR activation is detrimental, its moderate activation is beneficial for mediating repair of cutaneous wound and epithelial injury^41,42^. Because piperonylic acid caused mild EGFR activation and showed just a small amount of cytotoxicity in HaCaT keratinocytes (Supplementary Fig. S6), it could be effectively utilized for wound healing and skin rejuvenating cosmetic products. The effects of cosmetics for skin should be mild because cosmetics are applied for long period of time. Thus, piperonylic acid may be suitable for cosmetics with its activity to trigger mild keratinocyte regeneration. The smaller size of piperonylic acid may also be beneficial to permeate skin barrier more easily compared to EGF, which is 38.5 times bigger than piperonylic acid. Therefore, in conclusion, the EGF-like activity of piperonylic acid could be applied for skin health products and expected to be a successful EGF alternative.

## Methods

### Reagents and Antibodies

Dulbecco’s modified Eagle’s media (DMEM), Fetal bovine serum (FBS), Penicillin-Streptomycin, Trypsin-EDTA, Epidermal growth factor (EGF) (Peprotech), Piperonylic acid, Tyrphostin AG1478, Cyanogen bromide-activated-Sepharose^®^ 4B, Thiazolyl Blue Tetrazolium Bromide (MTT), Hoechst 33342 (Sigma-Aldrich), G418 (Calbiochem), Cell counting kit-8 (CCK-8) (Dojindo), Dako fluorescent mounting medium (Dako), CellROX^®^ Green Reagent (Life technologies), α-p-ERK T202/Y204, α-p-AKT S473, α-p-PLCγ1 Y783, α-ERK, α-AKT, α-PLCγ1, α-Egr-1 (CST), α-p-Tyr, α-EGFR, α-c-Fos, α-p-STAT3 S727, α-STAT3 (SantaCruz), α-FGFR2 (Abcam), α-GAPDH (Millipore), α-Actin (MP biomedicals), α-mouse IgG HRP conjugate (Thermo), and α-rabbit IgG HRP conjugate (Promega).

### Cell culture

HEK293A, A549, and HaCaT cell lines were cultured in DMEM supplemented with 10% FBS and 1% penicillin-streptomycin at 37 °C under 5% CO_2_ atmosphere. Prior to the treatment of compounds, cells were washed three times with phosphate buffered saline (PBS) and incubated in serum free DMEM for 24 hr.

### Biosensor assay

Modified biosensor vector was transfected into A549 cells using Neon^®^ transfection system (Invitrogen). The transfected cells were grown on glass chips in DMEM supplemented with 10% FBS and 1% penicillin-streptomycin for 24 hr. Cells were washed with warm PBS three times and the medium was exchanged with serum free DMEM. After 24 hr of serum depletion, cells were treated with compounds or vehicle (0.1% DMSO final) and incubated for appropriate time. Then, cells were fixed with 4% paraformaldehyde (PFA) for 40 min at room temperature (RT) and washed with PBS three times. Nucleus was stained with Hoechst (10 μg/ml) in PBS for 10 min and washed twice additionally. Cells were finally mounted with Dako fluorescence mounting medium. Images were captured using ZEISS fluorescent microscope.

### Western blot analysis

Cell lysate was obtained using a cell lysis buffer containing 50 mM Tris pH 7.5, 150 mM NaCl, 1 mM EDTA, and 1% Triton X-100. 20–30 μg of protein samples were separated by SDS-PAGE on 10% SDS gels and transferred to nitrocellulose membranes (110 V constant for 90 min, wet transfer). The membranes were labeled with primary antibodies for overnight at 4 °C and then labeled with horse radish peroxidase (HRP)-conjugated secondary antibodies. Chemiluminescence images were captured by ImageQuant LAS 4000 (Fuji).

### Piperonylic acid-4B pull-down assay

Piperonylic acid-4B and Control-4B beads were made using CNBr-activated Sepharose^®^ 4B (Sigma) according to the manufacturer’s instructions. Briefly, 1.25 mM of piperonylic acid or same volume of DMSO was added for coupling with 40 mg of CNBr-activated Sepharose 4B beads. For pull-down assay, 1 mg of HaCaT cell lysate was incubated with 30 μl of Control-4B or Piperonylic acid-4B bead for overnight at 4 °C. 500 μM of free piperonylic acid was added for competition. The bound protein was analyzed by Western blotting.

### Immunoprecipitation

Serum depleted HaCaT cells were treated with 100 μM of piperonylic acid or 1ng/ml of EGF for 10 minutes (same volume of DMSO for negative control). Harvested cells were lysed with cell lysis buffer that was previously mentioned. 500 μg of cell lysate was immunoprecipitated using anti-EGFR antibody with 25ul of protein-G agarose bead. The precipitated protein was analyzed by Western blotting. For analyzing FGFR2 activation, serum depleted cells were treated with 100 μM of piperonylic or 100ng/ml of FGF7 for 10 minutes, and anti-FGFR2 antibody were used for FGFR2 immunoprecipitation.

### Real time qRT-PCR

Total RNA was isolated with Ribospin^TM^-II RNA purification kit (GeneAll) according to the manufacturer’s instructions. Isolated RNA was reverse transcribed by ImProm^TM^-II Reverse Transcription System (Promega) according to the manufacturer’s instructions. For detection and quantification, the StepOnePlus^TM^ Real-Time PCR System (Applied Biosystems) was used. The sequences of the forward and reverse primers were as follows: *c-jun* forward 5′-AATAACACAGAGAGACAGACTTG-3′, reverse 5′-CTTGGATACCCTTGGCTTTAG-3′; *egr-1* forward 5′-TGACCGCAGAGTCTTTTCCT-3′, reverse 5′-TGGGTTGGTCATGCTCACTA-3′; *c-myc* forward 5′-TTCGGGTAGTGGAAAACCAG-3′, reverse 5′-CAGCAGCTCGAATTTCTTCC-3′; *c-fos* forward 5′-GTGTGTATTGTTCCCAGTGA-3′, reverse 5′-AGTTAATGCTATGAGAAGACTAAGG-3′; *β-actin* forward 5′-AGAGCTACGAGCTGCCTGAC-3′, reverse 5′-AGCACTGTGTTGGCGTACAG-3′.

### Cell growth assay

1 × 10^4^ HaCaT cells were plated with DMEM supplemented with 10% FBS and 1% penicillin-streptomycin on 96-well plate and were incubated for 24 hr. Following three washes with PBS, the medium was exchanged with compound or vehicle containing serum free DMEM (0.1% DMSO final). After 24 hr of additional incubation, Cell counting kit-8 (CCK-8) was treated as the manufacturer’s protocol and the optical density (OD) was read at 450 nm.

### UVB irradiation

1 × 10^4^ HaCaT cells were plated with DMEM supplemented with 10% FBS and 1% penicillin-streptomycin on 96-well plate and incubated for 24 hr. Cells were washed with PBS three times and thin layer of PBS was added. Cells were then exposed to UVB light (35 and 50 mJ/cm2) using 312 nm light source of CL-1000 (UVP). After UVB irradiation, cells were cultured in serum free DMEM with or without compounds for 24 hr. Control cells were identically processed but without UVB irradiation.

### Wound healing assay

2 × 10^5^ HaCaT cells were plated with DMEM supplemented with 10% FBS and 1% penicillin-streptomycin on 12-well plate. When cells reached confluence, a straight scratch per well was made using a yellow pipette tip. Following three washes with PBS, the medium was exchanged with compound or vehicle containing serum free DMEM (0.1% DMSO final). The medium was exchanged everyday just before the image was taken. Images were captured using Olympus phase contrast inverted microscope and the wound areas were quantified using ImageJ.

### Measure ROS generation

1 × 10^4^ HaCaT cells were plated with DMEM supplemented with 10% FBS and 1% penicillin-streptomycin on 96-well plate and incubated for 24 hr. Following three washes with PBS, the medium was exchanged with serum and phenol-red free DMEM and incubated for 24 hr. 10 minutes after compound treatment, 5 μM of CellROX^®^ reagent and 5 μg/ml of Hoechst were treated and incubated for 30 minutes. Then, the ROS signal images were captured using Olympus fluorescent microscope.

### Cell viability (MTT) assay

5 × 10^4^ HaCaT cells were plated with DMEM supplemented with 10% FBS and 1% penicillin-streptomycin on 96-well plate and incubated for 24 hr. Cells were washed with PBS three times and various concentrations of piperonylic acid were treated (0.1% DMSO final) in serum free DMEM. After 24 hr and 48 hr of incubation, cells were treated by Thiazolyl Blue Tetrazolium Bromide (MTT) (0.45 mg/ml final) and further incubated for 1.5 hr. The formazans were dissolved by 100 μl of DMSO and the OD was read at 570 nm.

### Statistical Analysis

All statistical analyses were performed using GraphPad Prism version 6.0. The significance of differences was assessed by the unpaired Student’s t–test or one-way ANOVA followed by the Tukey’s multiple comparison tests. A p-value of p < 0.05 was considered to represent a significance. All data are presented as mean ± SD or SEM.

## Electronic supplementary material

Supplementary information
