# Supplementary material for: Piperonylic acid stimulates keratinocyte growth and survival by activating epidermal growth factor receptor (EGFR)
Source: Sci Rep. 2018 Jan 9;8:162. doi: 10.1038/s41598-017-18361-3 (PMC5760518; doi:10.1038/s41598-017-18361-3)
Supplement: Supplementary file 1 — Supplementary information [file 41598_2017_18361_MOESM1_ESM.pdf]

Supplementary Information

Piperonylic acid stimulates keratinocyte growth and survival by activating epidermal growth factor receptor (EGFR)

Dohyun Lee[1], Jinsun Lim[1], Kyung-Chul Woo[3], Kyong-Tai Kim[1, 2, †]

[1]Department of Life Sciences, [2]Division of Integrative Biosciences and Biotechnology, Pohang University of Science and Technology, Pohang, 37673, Republic of Korea; [3]Newlife Cosmetics R&D Center for Skin Science, Gyeongsan, Gyeongbuk, Republic of Korea

[†]To whom correspondence should be addressed.

E-mail: **ktk@postech.ac.kr**

Supplementary Information

Supplementary Fig. S1

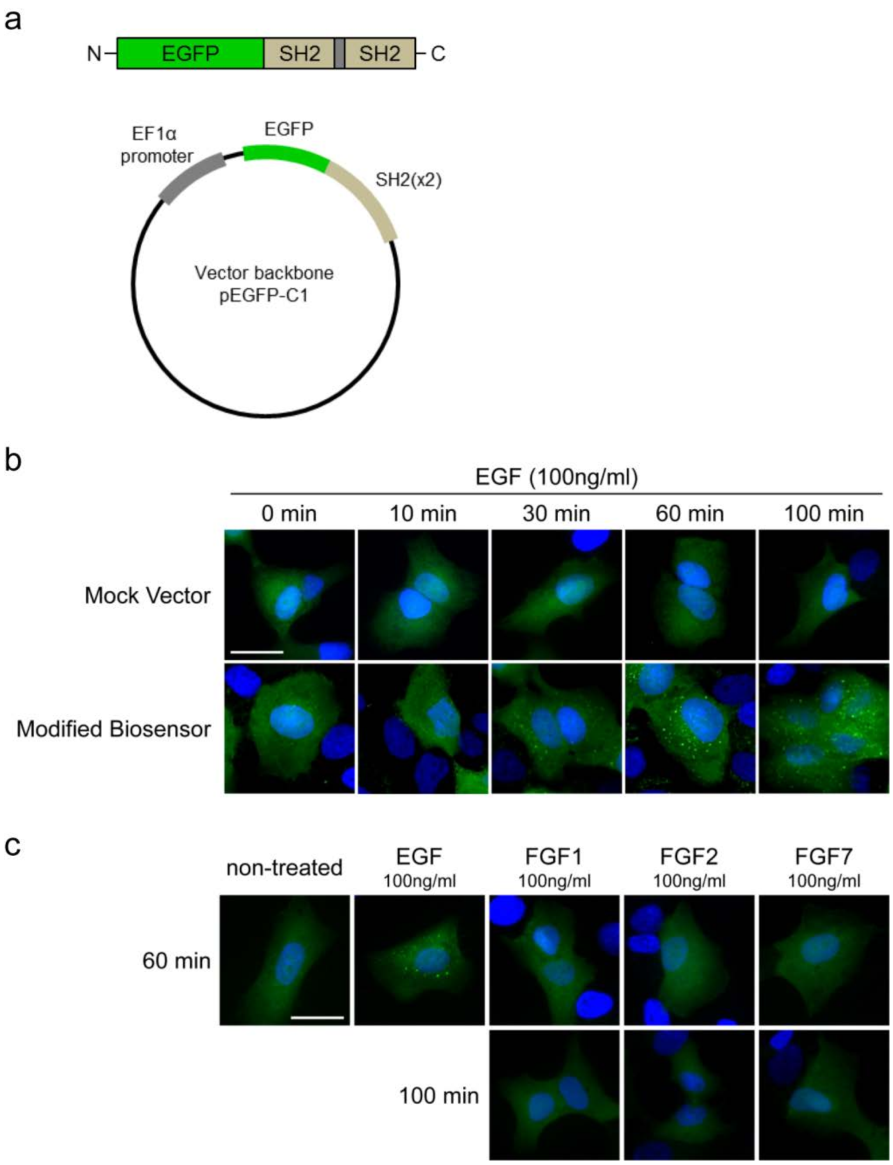

Supplementary Figure S1. (a) The structure of the modified biosensor vector system (bottom) and the biosensor protein (top). (b) The modified biosensor system detected EGFR activation shown in bright green fluorescent spots (Full version images of Fig. 1b). (c) The modified biosensor was quite sensitive to EGF compared to other Grb2 mediating growth factors. Scale bar, 50 ㎛. Blue fluorescence represents nucleus.

Supplementary Information

Supplementary Fig. S2

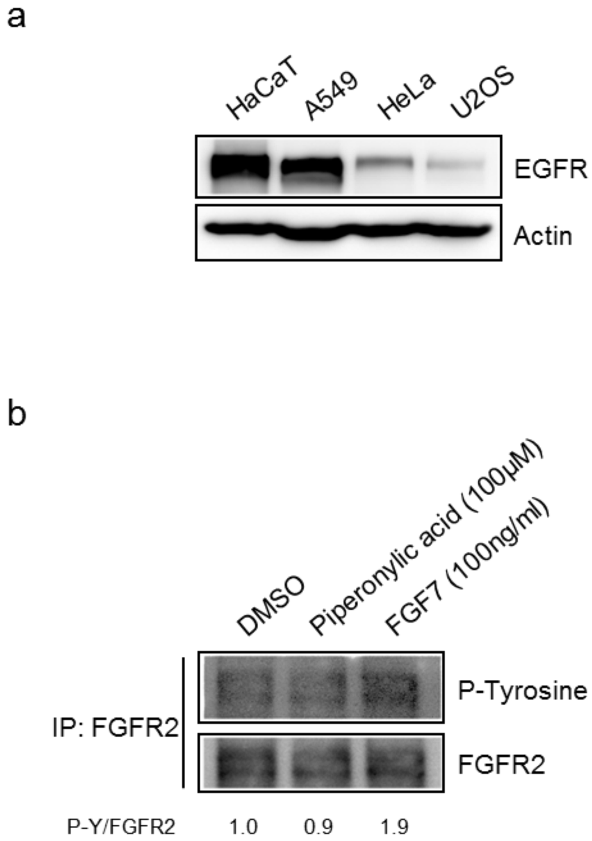

Supplementary Figure S2. (a) EGFR expression in various cell lines. HaCaT, skin keratinocyte; A549, alveolar basal epithelial adenocarcinoma; HeLa, epitheloid cervix carcinoma; U2OS, bone osteosarcoma. (b) FGFR2 was not activated by piperonylic acid treatment (10 minutes). FGF7, a ligand for FGFR2, was used as a positive control.

Supplementary Information

Supplementary Fig. S3

a

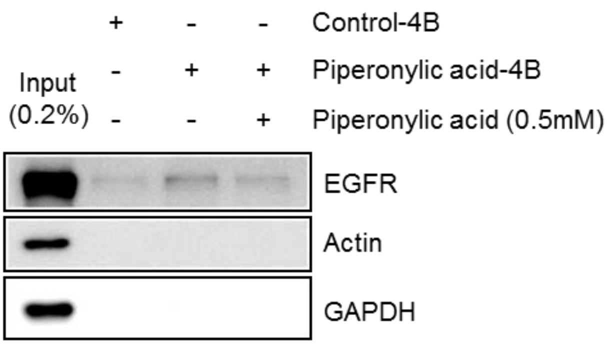

b

	Predicted binding site
Top score \ PDB entry	1MOX (A.A. 25-525)
1	K25, C251, R255
2	R334
3	S286, G305
4	Y270, P265
5	Y270
6	S286
7	R108
8	R255
9	C311, E317
10	L469

c

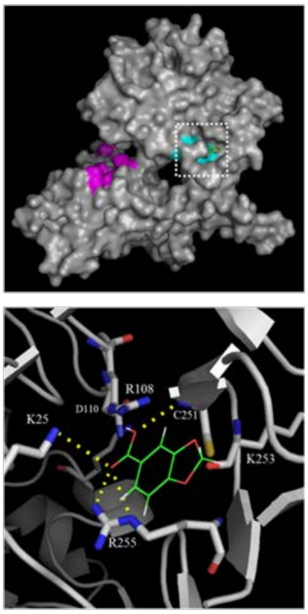

Supplementary Figure S3. (a) EGFR interacted with piperonylic acid-coupled Sepharose-4B. (b) A table of predicted binding sites of piperonylic acid on extracellular domain of EGFR (PDB entry 1MOX). Top 10 score docking models were selected by PatchDock. (c) Cartoons of the highest score docking model. Top, the docking pocket of extracellular domain of EGFR for piperonylic acid (Cyan) and for EGF (Magenta). Bottom, predicted polar contacts between piperonylic acid and EGFR at the docking pocket.

Supplementary Information

Supplementary Fig. S4

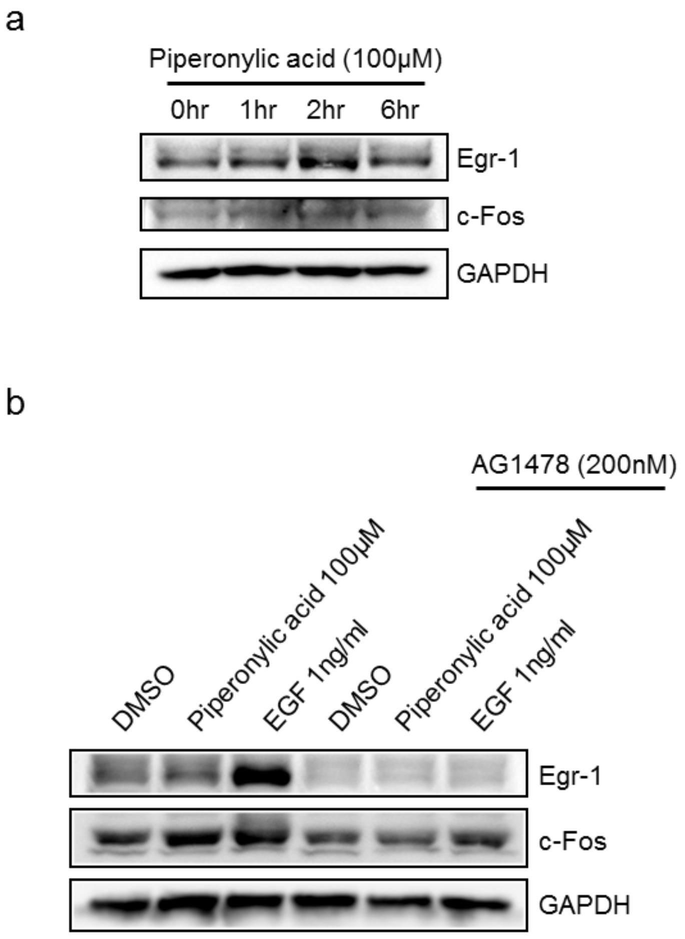

Supplementary Figure S4. (Related to Fig. 3a and b) (a) Time dependent Egr-1 and c-Fos protein expression changes by piperonylic acid (100μM) treatment. (b) Increased Egr-1 and c-Fos protein expression by piperonylic acid (treatment for 2hr) was blocked by AG1478.

Supplementary Information

Supplementary Fig. S5

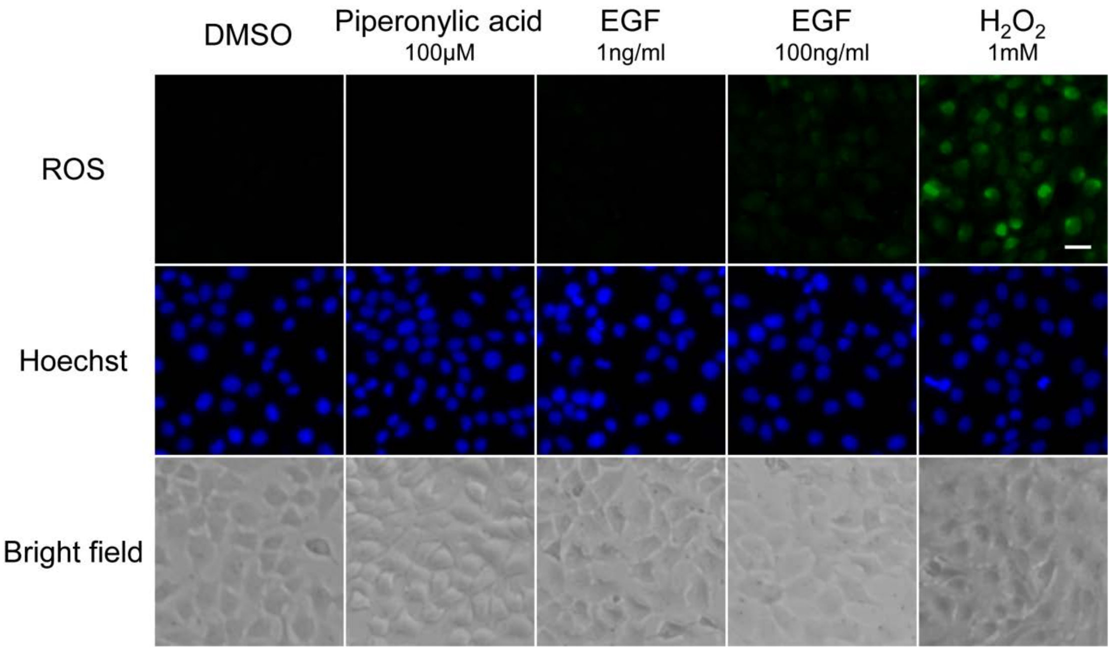

Supplementary Figure S5. ROS generation measured using CellROX® Green reagent. 100μM of piperonylic acid or 1ng/ml of EGF did not generate ROS upon 10 minutes treatment. Scale bar, 50 ㎛.

Supplementary Information

Supplementary Fig. S6

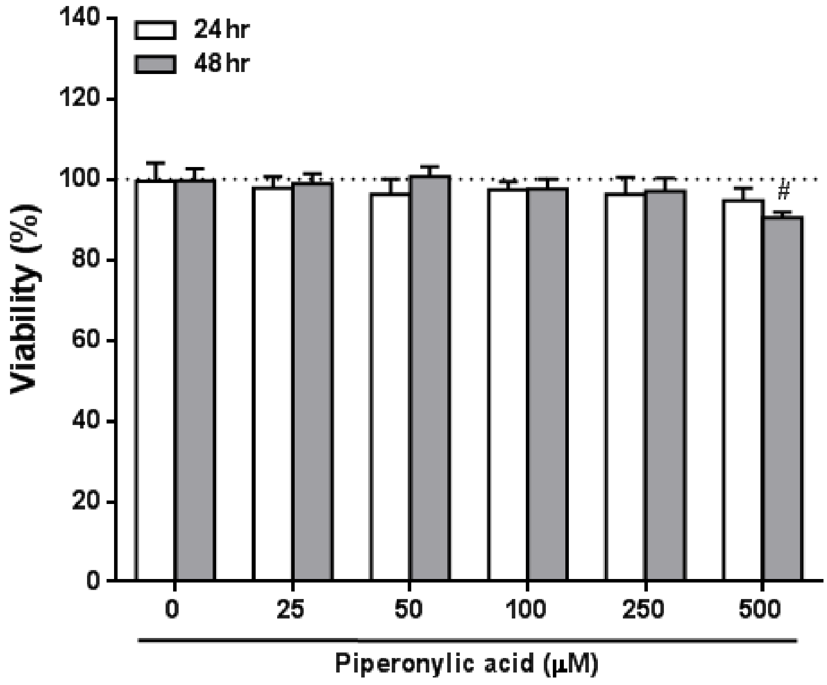

Supplementary Figure S6. Cytotoxicity of piperonylic acid in HaCaT cells measured by MTT assay. Data are presented as mean ± SD. Unpaired t-test. #p<0.0001.
